# Predicting student-teacher self-directed learning using intrinsic and extrinsic factors: a Theory of Planned Behavior adoption

**DOI:** 10.3389/fpsyg.2023.1211594

**Published:** 2023-09-14

**Authors:** Aukkapong Sukkamart, Paitoon Pimdee, Punnee Leekitchwatana, Watcharin Kongpiboon, Thiyaporn Kantathanawat

**Affiliations:** King Mongkut’s Institute of Technology Ladkrabang (KMITL), School of Industrial Education and Technology, Bangkok, Thailand

**Keywords:** extrinsic factors, intrinsic factors, pre-service teachers, self-directed learning, student teachers, Thailand, Theory of Planned Behavior

## Abstract

**Introduction:**

This study set out to develop a model that illustrates the causal relationship between factors influencing Thai student-teacher self-directed learning (SDL). To achieve this, the authors analyzed and applied the Theory of Planned Behavior (TPB) to investigate the role of family support, teacher support, friend support, fellow students, and the university in influencing SDL.

**Methods:**

The research used a sample of 468 student-teachers from five academic majors randomly selected from the King Mongkut’s Institute of Technology Ladkrabang (KMITL) Bachelor of Industrial Education Program during the 2021 academic year. The authors developed a questionnaire which was evaluated from both a panel of experts and a 30 student-teacher pilot test which found that item reliability was acceptable. LISREL 9.10 was used to analyze the data from the study’s structural equation model path analysis.

**Results:**

The results revealed that all the causal variables in the model positively influenced SDL, explaining the variance of influencing factors on SDL (R^2^) at 51%. SDL comprised five variables, when ranked in order of importance these were fellow students (FSt), teacher support (TS), family support (FS), friend support (FrS), and their university (Uni). The study also highlighted issues concerning each student-teacher’s learning time management ability and their perception of the university’s ability/willingness to allow each individual to choose their course, thus enhancing their SDL learning ability skills.

**Discussion:**

It was speculated that some university educators even today perceive SDL as an adult education tool which they perceive is not appropriate at the university level. The authors also felt that for younger student-teachers that the COVID-19 medical emergency limited teacher/student/university interaction, thus contributing to student misconceptions about support availability. This study contributes significantly to the literature by investigating how TPB intrinsic and extrinsic factors impact a university student’s self-directed learning.

## 1. Introduction

The COVID-19 pandemic has significantly impacted how people learn around the world, including in Thailand. In particular, higher education in Thailand during the pandemic shifted to fully online teaching and learning (UNESCO, 2020). As a result, the need for online teaching has increased, and education management at the higher education level has focused on enhancing learners’ ability to search for and acquire knowledge on their own (Rattanaarun et al., 2023). This includes self-directed learning (SDL), which emphasizes learners being proactive and passionate in pursuing knowledge, setting goals, and developing their learning strategies (Kerdtip and Angkulwattanakit, 2023). Self-directed learners are determined and persistent in achieving their goals and will continue to learn until they reach the knowledge, understanding, skills, and competency they desire (Bergamin et al., 2019).

Therefore, this study highlights the importance of the creation of open learning environments in which students gain access to information and knowledge more readily. The study also determines ways to allow students to gain access to resources which were economical and affordable. The study further highlights the need for change implementation and the creation of new learning styles to keep up with the pace of global information and technological changes. The study also fills the research gap by showing how student self-learning is influenced by various factors including what is around them and how it affects them.

Support for these ideas and objectives can first be found from studies by Maphalala et al. (2021) and Morris (2019), in which SDL ability was viewed as essential for successful online teaching, especially in a rapidly changing technological world. SDL also refers to the ability of individuals to take responsibility for their learning, which includes determining their learning objectives, content, and progress, choosing learning methods and techniques, tracking their learning process, and evaluating their learning outcomes (Deci and Ryan, 1985). SDL is related to the desire and ability of learners to make their own choices about their learning and lifelong learning skills (Tekkol and Demirel, 2018), and it is believed to be essential for allowing learners to explore and build knowledge independently. Self-determination theory suggests that purposeful and active learning behaviors can produce more satisfactory learning outcomes (Reeve, 2012). SDL thus enables self-management and self-evaluation, influencing the future development and spread of online education (Sun et al., 2022).

Self-learning refers to an individual’s initiative in identifying their own learning needs, setting goals, and choosing preferred sources to learn from. This process also includes selecting appropriate learning strategies and evaluating outcomes, with or without external assistance (Knowles, 1975). In self-learning, individuals are responsible for their learning, with the responsibility shifting away from external sources such as teachers.

This is consistent with other studies, such as Prapatsaranon et al. (2022), who determined that for Thai university students in their self-learning promotion, the ability to choose their content or types of activities was viewed as most essential. Another study by Renninger and Bachrach (2015) also supported this, which determined that participatory behavior willingness involved the ability to self-select interesting content or activity types, followed by participation in learning activities.

Active involvement and control by learners are essential in this process. Self-guided learning involves conceptualizing, designing, implementing, and assessing learning based on student guidance, also referred to as a learner-controlled learning method. Moreover, self-learning is a goal that individuals strive to achieve, accepting personal independence and preferences as part of the process.

Recent technological advances have significantly impacted education, providing students with more opportunities to learn independently and create their own learning experiences (Bond and Bedenlier, 2019; Pan, 2020). Emerging technology-based learning ecosystems, such as online, e-learning, m-learning, and informal technology learning approaches, have become more widespread and offer many advantages (Wongpratoom and Sranamkam, 2019; Klein-Collins and Travers, 2020; Gupta and Singh, 2022, p. 331). However, despite the benefits, there are still challenges in effectively using technology to facilitate self-learning and its use in appropriate Internet use behavior (Pimdee and Leekitchwatana, 2022). External support is also required, such as teacher influence and school factors, to enhance the use of technology for learning. Studies have found that a teacher’s influence can affect students’ decisions to use technology in the self-learning process.

This research uses the Theory of Planned Behavior (TPB) to explain how student-teachers learn independently (Tzeng et al., 2022, p. 2). TPB was created by Ajzen (1985) and expanded in later years (Ajzen, 1991; Kotahwala, 2020). Using the TPB as a model is a helpful way of explaining behavioral intention, which is the Theory of Reasoned Action (TRA) expanded, which reasons that any factor can indirectly influence use behavior through attitude and subjective norms (Madden et al., 1992). The TPB Theory is also a social psychology theory developed from TRA. It contends that manifestations of human behavior are guided by three beliefs: behavioral beliefs (student variable), normative beliefs (family, friends, and teacher variables), and beliefs, and about control beliefs (University variable), where each belief affects different variables.

Therefore, this study looks at two types of sources that influence student-teachers: external or extrinsic factors (e.g., family, friends, teachers, and universities) and internal or intrinsic factors (e.g., motivation and planning) (Schweder and Raufelder, 2021). Moreover, according to multiple studies, intrinsic motivation occurs when three “basic psychological needs” are met (Deci and Ryan, 1985, 2013; Ryan and Deci, 2017). These BPNs are autonomy, competence, and social relatedness, with autonomy being a student teacher’s willingness to self-determine their actions (Deci, 1992). Also, Yeager et al. (2017) have added that autonomy is “a core value for human motivation,” while Yeager et al. (2017, p. 437) added that autonomy provides people with a “sense of free will, freedom of choice, and self-reliance.”

Education technology has transformed learning into a more self-directed process (Tzeng et al., 2022, p. 2). This has been facilitated by the vast array of digital devices and operating environments and opportunities for collaborative learning (Sarnok et al., 2019; Wongpratoom and Sranamkam, 2019). Against this background, Tzeng et al. (2022, p. 2) have stated that research is now heading from extrinsic to intrinsic motivation factors (IMF), with IMFs related to social cognitive theory (SCT) factors gaining more attention. Additionally, SCT is now combined with TPB (Shih, 2008).

Extrinsic is represented by the results from outside student factors (family, friends, teachers, and university variables), while intrinsic results are represented by inside student factors (Student variable). Extrinsic motivation is motivation that comes from external things or factors, outside the person. Extrinsic motives are usually exhibited by reward-driven behavior, which can be considered a form of behavioral modification (Klotz et al., 2012). Moreover, usually extrinsic motives are used to achieve results that a person does not get from intrinsic motivation (Ryan and Deci, 2017).

Therefore, the research aims to see if these factors affect how student-teachers learn and to understand the relationship between self-learning and student teachers. The study is critical because it helps us understand what influences self-learning in student-teachers and how we can help them learn better.

### 1.1. Research objectives

1. The authors of this study have established research objectives that involve the use of the Theory of Planned Behavior (TPB) and concepts of intrinsic and extrinsic factors to develop a path-analysis model of the factors that affect student-teacher self-directed learning (SDL).
2. Once the model’s constructs are identified, the authors will conduct a goodness-of-fit (GoF) analysis and a confirmatory factor analysis (CFA) to determine the model’s fit before moving on to a structural equation model (SEM) analysis.
3. In order to gather data, the authors will develop and assess a questionnaire before conducting an online survey to gather the opinions of Thai student-teachers on what they perceive to influence their SDL abilities.

## 2. Literature review

This section contains an overview of the theory and literature related to the intrinsic and extrinsic variables for the study, including the student-teacher’s *family support, teacher support, friend support fellow students*, their *university*, and *self-directed learning*.

### 2.1. Family support (FS)

Nurrokhmanti et al. (2016) studied the external factor of motivation and its usefulness as an evaluation method for Indonesian medical school, and discovered there was a significant relationship between motivation and SDL readiness. Paiwithayasiritham (2013) studied 400 Thai university students’ SDL characteristics and determined that the most important was each student’s learning opportunity, followed by their learning fondness and their concept of being an effective learner. However, gender and year of study were determined not to affect SDL.

Jouhari et al. (2015) investigated which factors affect self-regulated learning (SRL) in medical students. Results showed that five themes affected SRL, including the student’s family and ability to supervise and support the student, their peers, their teachers, the educational environment, and each student’s facilitating and inhibiting personal factors.

Therefore, after a review of the literature concerning student-teacher *family support*, the authors wish to propose the following two hypotheses:

*H1*: Family support influences self-directed learning in a direct and positive manner.

*H2*: Family support influences fellow students in a direct and positive manner.

### 2.2. Teacher support (TS)

Teachers play a critical role in fostering SDL in students as they guide and support students in developing the skills, attitudes, and behaviors necessary to become effective learners. By creating a supportive and engaging learning environment, teachers can help students develop autonomy, self-motivation, and critical thinking skills, essential for SDL. Teachers can also act as mentors and coaches, providing students with feedback and guidance on their progress, helping them set goals, and providing opportunities to reflect on their learning (Rodrangsee et al., 2022).

Technology in all its forms plays a critical role in facilitating SDL, as it provides students with access to unlimited resources and information to support their learning goals (Tzeng et al., 2022). Technology can also enable students to learn at their own pace, allowing them to work through content at a comfortable speed (Khan, 2016; Beach, 2017).

According to Pan and Chen (2021), teacher behavior support (TBS) involves a teacher’s organization and management support capacities which assist learners in participating in tasks and activities using technology. Hoi and Mu (2021) in Vietnam added that TBS can also significantly influence technological devices’ perceived ease of use (PEU). Gallivan et al. (2005) added that coworkers significantly influence ICT (information communication technology) usage, whereas individual-level factors exhibit more modest effects.

Pan and Chen (2021) sampled the opinions of 197 undergraduate students in Eastern China concerning how teacher support influenced their language SDL outside the classroom. Results revealed the importance of increasing teachers’ awareness in enhancing students’ language SDL outside the classroom. Equally, the study indicated the essential nature of using technology. Therefore, teachers play an essential role in determining the student’s learning experience quality and their cognitive learning behavior, self-learning skills, and social behavior (Farmer et al., 2011).

After a review of the literature concerning student-teacher *teachers*, the authors wish to propose the following three hypotheses:

*H3*: Teacher support influences fellow students in a direct and positive manner.

*H4*: Teacher support influences the university in a direct and positive manner.

*H5*: Teacher support influences self-directed learning in a direct and positive manner.

### 2.3. Friend support (FrS)

The influence of friends on SDL has been discussed in the academic literature extensively, and it has been suggested that peers can play a crucial role in promoting self-directed learning in students (Sidianto et al., 2022, p. 1693).

One way that friends can influence self-directed learning is through peer modeling. When students observe their peers engaging in self-directed learning behaviors, such as seeking out information independently or managing their time effectively, they are likelier to adopt these behaviors (Zimet et al., 1988). Peer support and encouragement motivates students to use SDL and pursue their academic goals.

Another way that friends can influence SDL is through collaborative learning activities. When students work together in groups or pairs, they can share knowledge and resources and engage in problem-solving activities that require them to use SDL strategies. Collaborative learning can foster a sense of responsibility for learning and help students develop the skills and confidence they need to engage in SDL independently.

Sheasby and Smith (2023) conducted a study to examine the relationship between predictor variables and pro-environmental behavior. The significance of this relationship was assessed using multiple linear regression. The study found that the influence of others had the most significant impact on pro-environmental behavior, followed by an individual’s environmental responsibility (ER) score. The ER score was assessed using a series of graded statements that measured an individual’s actions toward environmental responsibility. Overall, the literature suggests that peer influence can be influential in promoting SDL among students and that teachers and educators can leverage this influence to support and enhance students’ SDL skills.

After a review of the literature concerning student-teacher *friend support*, the authors wish to propose the following three hypotheses:

*H6*: Friend support influences fellow students in a direct and positive manner.

*H7*: Friend support influences the university in a direct and positive manner.

*H8*: Friend support influences self-directed learning in a direct and positive manner.

### 2.4. University (Uni)

According to Leatemia et al. (2016), the availability of learning facilities and the academic atmosphere in the academic environment can significantly affect a student’s level of SDL readiness. Similarly, Huang’s (2008) research also suggests that a supportive learning environment, as perceived by students, significantly impacts their SDL readiness. In addition to these internal factors, external factors can play a crucial role in SDL readiness. Nurrokhmanti et al. (2016) identify various external factors, including support from family and friends, university facilities, problems encountered, peer relationships, and parent and friend influence in the learning environment, that can significantly affect a student’s SDL readiness.

Therefore, after a review of the literature concerning the student-teacher *university*, the authors wish to propose the following hypothesis:

*H9*: The university influences Self-Directed Learning in a direct and positive manner.

### 2.5. Fellow students (FSt)

Knowles (1975, p. 18) stated that SDL is a process in which student take the initiative, either without or with the help of others. From their imitative they develop how to recognize their learning needs and strategies, how to formulate their learning goals, how and where to find learning resources, and how to assess their own learning results.

These concepts align with the research conducted by Pan (2020), which demonstrated a correlation between learner technology acceptance, technological self-efficacy, and their attitude toward technology-based SDL. The study results also revealed the importance of learning motivation and SDL in technological environments. These findings are similar to a study from Turkey in which Durnali (2020, p. 129) reviewed how 835 students’ SDL skills and online learning were affected during the COVID-19 pandemic and commented on the importance of perceived leadership in a learner’s self-regulation and engagement in an online learning environment.

These ideas also align with Ramli et al. (2018) who reported that internal factors such as achievement motivation, interest in learning, and academic self-concept positively impact SDL readiness. Nyambe et al. (2016) also reported on student internal factors and motivation and stated they were crucial to SDL readiness. Students with high levels of achievement motivation, interest in learning, and positive academic self-concept are more likely to manage their study time effectively and independently seek academic information from various sources. Their desire for achievement and love of learning motivates them to pursue their goals independently. Furthermore, Saeid and Eslaminejad's (2017) research showed a significant correlation between SDL readiness and students’ self-efficacy and achievement motivation.

On another level, various studies have voiced their concerns for student well-being due to the effects of the COVID-19 pandemic. In South Africa, Van Tonder et al. (2022) felt the need to establish a learner-self-directed academic and personal well-being program as an education priority. Moreover, the authors stated that exposure to an intervention process holds benefits for equipping teachers with teaching strategies that enable classroom conditions that support the development of students’ self-regulated thinking skills and self-directing academic and personal well-being.

Therefore, after a review of the literature concerning *student support*, the authors wish to propose the following hypothesis:

*H10*: Fellow students influence self-directed learning in a direct and positive manner.

### 2.6. Self-directed learning (SDL)

Self-directed learning (SDL) is an essential aspect of professional development for student teachers, which refers to the ability to plan, implement, and evaluate one’s learning goals and progress (Pimdee et al., 2023). Moreover, there has been an ever-growing interest in the literature on the importance of SDL for student teachers. SDL has also been identified as an essential element in lifelong learning (Tekkol and Demirel, 2018; Salleh et al., 2019; Loeng, 2020), with Tough being one of the first scholars to put forward a comprehensive description of SDL (Loeng, 2020). Moreover, Tough observed that adults spend a remarkable amount of time on learning projects meant to acquire additional skills for their advancement (Tough, 1978, p. 250). Therefore, it was stated at the time that learning can be performed through reading, listening, observation, course participation, reflection, exercise, or otherwise.

SDL is a process that involves individuals taking responsibility for their learning, including identifying their learning needs and goals, seeking out resources, and evaluating their progress. This concept has been recognized and researched for decades, with scholars emphasizing the importance of learner autonomy and decision-making in the SDL process. Brookfield (1995) highlights the role of learners in making decisions about what to learn, when, how much to learn, and whether they have learned it well enough. Sze-Yeng and Hussain (2010) emphasize the personal attributes of learners in promoting learner autonomy.

As defined by Brockett and Hiemstra (2018) and Knowles (1975), SDL is a learning process that involves planning, goal-setting, seeking and selecting resources, and evaluating the learning process. Barnes et al. (2007, p. 2) have emphasized that the Net Generation requires SDL opportunities, interactive environments, various forms of feedback, and assignment choices that utilize diverse resources to create personally meaningful learning experiences. Learners need to learn how to develop their SDL abilities actively without being solely taught by others.

The literature supports that SDL is crucial in developing student teachers’ effectiveness as educators. According to Pimdee et al. (2023), SDL enables student teachers to acquire the skills necessary to work independently and take responsibility for their learning, which is essential for their professional growth.

In addition, SDL helps student teachers to develop critical thinking and problem-solving skills. These skills are essential for educators because they need to be able to assess complex situations and make informed decisions that benefit their students’ learning outcomes. Furthermore, the ability to think critically and solve problems is essential in addressing the challenges of the rapidly changing education sector.

Overall, research suggests that SDL is critical in developing student teachers’ effectiveness as educators. By promoting the development of crucial skills such as autonomy, responsibility, critical thinking, and problem-solving, SDL can help student teachers become more adaptable and successful in their careers. Indeed, studies have highlighted the importance of using SDL strategies to facilitate SDL among student teachers. These strategies can enhance the effectiveness of SDL and enable student teachers to take greater ownership of their learning.

One of the most critical SDL strategies is goal-setting, which involves setting clear, specific, and achievable goals. By setting goals, student teachers can identify the learning objectives they want to achieve and develop a plan for achieving them. Goal-setting can help student teachers to stay focused, motivated, and accountable for their learning.

Reflection is another crucial SDL strategy that enables student teachers to evaluate their progress, identify their strengths and weaknesses, and determine the steps they need to take to improve their learning outcomes. Reflection can help student teachers to develop a deeper understanding of their learning process and increase their self-awareness.

Feedback is also a key SDL strategy that provides student teachers with information on their progress and areas for growth. Feedback can come from peers, mentors, or instructors and can be formal or informal. Feedback helps student teachers to understand their performance and identify specific areas for improvement.

SDL strategies such as goal-setting, reflection, and feedback can facilitate SDL among student teachers. These strategies can enhance the effectiveness of SDL and enable student teachers to become more independent and successful learners.

Furthermore, research has shown that a supportive learning environment is essential for promoting SDL among student teachers (Camacho and Legare, 2016). A supportive learning environment can be created by providing resources and opportunities for professional development and by providing opportunities for collaboration and networking. Moreover, SDL is interconnected with personalized learning and *competency-based education* (CBE) in non-traditional higher education, which helps educators serve the needs of employers after students graduate (DeMink-Carthew et al., 2017; Williamson and Williamson, 2020). Furthermore, SDL plays a critical in problem-based learning (PBL), which is a student-centered approach to education (Abdullah et al., 2019) which focuses on permitting students to solve open-ended problems (Leary et al., 2019).

To elaborate further, metacognition refers to the learner’s ability to monitor and regulate their thinking and learning processes, while motivation refers to the learner’s drive or interest in learning (Long 2000, p. 15). Self-regulation involves the learner’s ability to plan, monitor, and evaluate their learning, while choice refers to their ability to decide what and how they learn. Competence refers to the learner’s belief in their ability to learn and perform, while control refers to the learner’s ability to manage their learning environment. Finally, confidence refers to the learner’s belief in their capacity to learn and succeed.

According to capability theory, the development of these capabilities is vital for self-directed learning to occur. However, the learner must have a reason to value the development of these capabilities. This highlights the importance of intrinsic motivation, which stems from the learner’s interests, values, and goals, as opposed to external rewards or pressure. The learner must believe that developing these capabilities will help them to obtain their goals and fulfill their needs (Van der Walt, 2016).

In conclusion, SDL is an essential aspect of professional development for student teachers, and the use of SDL strategies and a supportive learning environment can facilitate the development of SDL in student teachers.

## 3. Materials and methods

### 3.1. Population and sample

The research was conducted on undergraduate students enrolled in the Industrial Engineering program at the School of Industrial Education and Technology at the King Mongkut’s Institute of Technology Ladkrabang (KMITL) in Bangkok, Thailand, during the academic year 2021. The total population was 1,749 students (King Mongkut’s Institute of Technology Ladkrabang (KMITL), Office of the Registrar, 2016). To determine the appropriate sample size, the authors followed the suggestions of Schreiber et al. (2006) and Nicolaou and Masoner (2013), who recommended a sample size of approximately 20 times the number of observed variables in the model. Since this research involved 23 observed variables, a targeted sample size of 500 students accounted for potentially incomplete questionnaires (Pimdee, 2020). Simple random sampling was used to select the study’s 468 student-teachers from each major, ensuring that the sample size for each major was proportional to the population size, as shown in Table 1.

**Table 1:** The population and samples are classified by gender and academic programs.

Academic programs	Population			Collection process					
				Sample target			Actual sample		
	Male	Female	Total	Male	Female	Total	Male	Female	Total
Agricultural education	112	238	350	32	68	100	43	55	98
Engineering education	308	220	528	88	63	151	75	72	147
Architectural education	87	172	259	25	49	74	21	47	68
Design education	96	208	304	27	59	87	31	53	84
Interior environmental design	77	231	308	22	66	88	17	54	71
Total	680	1,069	1,749	194	306	500	187	281	468

### 3.2. Research tools

The tool used to collect data was a seven-part questionnaire. Part 1 contained items related to each student-teachers personal and academic life (See Table 2). Parts 2–7 contained items related to the 23 observed variables identified for the six constructs. Student-teacher opinions were evaluated using a five-level opinion scale in which 4.50–5.00 indicated that they *strongly agreed*, 3.50–4.49 as *somewhat agree, 2*.50–3.49 was *moderate agreement*, 1.50–2.49 was *disagree*, and finally, 1.00–1.49 was *minimal agreement* (Yurayat and Tuklang, 2023, p. 75).

**Table 2:** Student-teacher respondent general characteristics (*n* = 468).

Items	Category	Number	%
Gender	Male	187	39.96
	Female	281	60.04
Academic major	Agricultural education	98	20.94
	Engineering education	147	31.41
	Architecture education	68	14.53
	Design education	84	17.95
	Interior environment design education	71	15.17
Current cumulative GPA	Between 2.00 and 2.49	31	6.62
	Between 2.50 and 2.99	133	28.42
	Between 3.00 and 3.49	225	48.08
	Between 3.50 and 4.00	79	16.88

Furthermore, Part 2 contained four items related to each student-teacher’s home and *family* life, Part 3 contained four items related to how *teachers* influenced each individual’s SDL, Part 4 contained four items about how each student-teacher’s friends influenced each individual’s SDL, Part 5 contained three items related to each student’s SDL including their self-management, self-control, and learning desire (Pimdee et al., 2023), Part 6 contained three items related to how the university (KMITL) influenced their SDL, and finally, Part 7 contained five items about each student’s SDL aspirations and processes. Each part’s items are detailed in Table 3.

**Table 3:** Descriptive statistics of study variables.

Variables	*n*	Mean	SD	Skewness	Kurtosis
Family support (FS)	468	4.12	0.65	−0.75	1.48
Teacher support (TS)	468	3.80	0.70	−0.56	0.60
Friend support (FrS)	468	4.03	0.74	−1.11	2.61
Fellow students (FSt)	468	4.07	0.54	−0.39	1.66
University (Uni)	468	3.52	0.80	−0.64	0.45
Self-directed learning (SDL)	468	4.01	0.46	−0.48	3.43

However, before the actual distribution of the survey, a pilot test was undertaken in 2021 using the questionnaire and 30 KMITL student-teachers who were not part of the final study (Taherdoost, 2016). Analysis of the results used discriminant power (discrimination) based on a corrected index of Item-Objective Congruency (IOC) and confidence (reliability) and by Cronbach’s alpha coefficient method (Table 4; Ditsuwan and Sukkamart, 2022).

**Table 4:** Reliability and discrimination of the latent variables.

Latent variables	Items	Discrimination	Cronbach α (*n* = 30)
Home/family	4	0.62–0.82	0.86
Teachers	4	0.55–0.67	0.80
Friends	4	0.75–0.89	0.91
Self-directed learning	24 (3 aspects)	0.36–0.68	0.91
University	3	0.71–0.87	0.87
Student	5	0.60–0.79	0.86

### 3.3. Data collection

The researchers initially set a target sample size of 500 based on multiple scholars who have suggested that robust samples should include 20 questionnaires for each observed variable (Schreiber et al., 2006; Nicolaou and Masoner, 2013) as the final questionnaire contained 23 observed variables, 460 completed questionnaires were needed. However, knowing some questionnaires might be incomplete, the number was rounded to 500 and set as the target.

As the COVID-19 virus was still active and social distancing requirements were still in place in 2021, the researchers distributed their questionnaire using Google Forms. Assistance was obtained from a team of research assistants and coordination through a network of student advisors in each subject area and grade level. A total of 468 completed questionnaires were returned, accounting for 93.60% of the desired target sample (*n* = 500), which was considered sufficient (Table 1).

### 3.4. Data analysis

The researchers analyzed the data using ready-made statistical programs. The details of the analysis are as follows.

1. General data were analyzed using descriptive statistics and the SPSS for Windows Version 21 program.
2. Confirmatory Factor Analysis (CFA) of the external latent variables and internal latent variables used LISREL 9.10 using the goodness-of-fit (GoF) criterion detailed in Table 5.
3. Model validity and the effect size between the variables in the student-teacher SDL model were analyzed using a LISREL 9.10 latent variable path analysis and goodness of fit (GoF) statistics, as detailed in Table 5. As the calculated statistics passed the stated criteria, this indicated that the causal relationship model had good validity and the model was in good agreement with the empirical data.
4. The experts’ and student-teacher pilot-test opinions concerning the draft questionnaire items were analyzed using S*PSS* for Windows Version 21. Item validity was accomplished with the assistance of five experts learning management, research and measurement/evaluation who rated item validity using the index of item-objective congruence developed by Rovinelli and Hambleton (Turner and Carlson, 2003). In this study’s case, all IOC values were between 0.60 and 1.00.
5. The interpretive mean criteria used 4.50–5.00 to indicate that they had *strong agreement,* followed by 3.50–4.49 indicting *high agreement,* 2.50–3.49 indicting *moderate agreement*, 1.50–2.49 indicting *low agreement*, and 1.00–1.49 indicating *no agreement* (Yurayat and Tuklang, 2023, p. 75).
6. Data analysis was then undertaken using descriptive statistics, including the mean, standard deviation (SD), frequency, and percentage.

**Table 5:** Results of component analysis of latent and internal-external variables.

Constructs	Items	Std. factor loading	*R*^2^	AVE	CR
Family support (FS)	FS1	0.73	0.54	0.60	0.85
	FS2	0.70	0.36		
	FS3	0.71	0.51		
	FS4	0.93	0.86		
Teacher support (TS)	TS1	0.73	0.53	0.61	0.86
	TS2	0.76	0.58		
	TS3	0.81	0.66		
	TS4	0.83	0.68		
Friend support (FrS)	FrS1	0.91	0.83	0.73	0.92
	FrS2	0.89	0.79		
	FrS3	0.82	0.67		
	FrS4	0.80	0.64		
Self-directed learning	SDL1	0.79	0.62	0.80	0.92
	SDL2	0.99	0.99		
	SDL3	0.89	0.79		
University (Uni)	Uni1	0.73	0.54	0.65	0.85
	Uni2	0.86	0.64		
	Uni3	0.82	0.67		
Fellow students (FSt)	FSt1	0.73	0.54	0.54	0.85
	FSt2	0.69	0.47		
	FSt3	0.73	0.54		
	FSt4	0.77	0.59		
	FSt5	0.75	0.56		

## 4. Results and discussion

### 4.1. Student-teacher respondent characteristics

First, it should be noted that in many higher education institutions throughout Southeast Asia, there is a significant imbalance between enrolled male and female students, especially in Thailand and Malaysia (Arttachariya, 2012; Study International News, 2015; Pimdee et al., 2023). In this study, there was no exception, as 60.04% identified as female, while the remaining 39.96% identified as male (Table 2). Also, this study used five academic majors for the sample, with engineering education and agricultural education representing 31.41 and 20.94%, respectively. Finally, most student-teachers (48.08%) maintained a GPA range of 3.00–3.49 even during the COVID-19 online learning under regiment, which is now referred to as part of the *New Normal* in Thailand (Srakaew et al., 2021; Pimdee et al., 2023).

### 4.2. CFA results

This component analysis was undertaken on both the external and internal latent variables using a confirmatory factor analysis (Table 3). Shrestha (2021) has suggested that Cronbach’s Alpha *α* values should be ≥0.70. As such, this study is α values for the latent variables were 0.80–0.91, indicating acceptable internal consistency and scale reliability for the questionnaire items (Daengneam et al., 2023, p. 83).

Table 3 also shows each latent variable’s average variance extracted (AVE) and composite reliability (CR) values. Multiple studies have suggested that CR values ≥0.7 are acceptable. The authors note that the AVE value for *students* is seemingly low at 0.54 (≤ 0.5). However, Fornell and Larcker (1981) have suggested that when the CRs are ≥0.6 and the AVEs are ≥0.5, values as low as 0.4 can be accepted. Therefore, the analysis shows strength.

Furthermore, Chuenban et al. (2021) have stated that *R*^2^ values should not be ≤0.20. Factor loadings should also be ≥0.5. In statistics, the coefficient of determination is often denoted as R-squared or R^2^, which is a measure that represents the proportion of the variance in the dependent variable (the variable the study is trying to predict) that is explained by the independent variables (the predictors) in a regression model. In simpler terms, it tells you how well the independent variables explain the variability of the dependent variable. Composite/construct reliability (CR) should be ≥0.7. After these tests, it is recommended that the model’s fit validity should be tested by use of an AVE ≥ 0.5. Given these criteria for AVE, CR, loading, and *R*^2^, it was determined that all latent and observed variables were within range of the acceptance criteria.

### 4.3. Correlation coefficient analysis

Table 5 details the correlation coefficient analysis results of the six latent variables (Pan, 2020).

### 4.4. Causal relationship model analysis results

The analysis of relationships between 23 variables by a Pearson product–moment correlation (PPMC) (r) analysis (Table 6). Results from the 23 PMMC indicator testing showed that the construct validity (CV) for the final model’s variables was correlated and in the same direction. Additionally, Chuenban et al. (2021) have suggested that *r* value strength interrelationship interpretation is as follows: +/−0.10–0.29 is weak, +/−0.30–0.49 is moderate, and +/−0.50–1.00 is strong.

**Table 6:** Correlation coefficients between latent variables.

Latent variables	FS	TS	FrS	FSt	Uni	SDL
Family support (FS)	**(0.77)**					
Teacher support (TS)	0.48**	**(0.78)**				
Friend support (FrS)	0.50**	0.42**	**(0.85)**			
Fellow students (FSt)	0.57**	0.51**	0.54**	**(0.73)**		
University (Uni)	0.36**	0.62**	0.37**	0.33**	**(0.81)**	
Self-directed learning (SDL)	0.53**	0.54**	0.50**	0.72**	0.38**	**(0.89)**

**Sig. ≤ 0.01, bold values on the diagonal are the square roots of AVE. To establish the discriminative validity, the value should be greater than the inter-construct correlations.

Table 6 also shows that the correlation coefficients among all observed variables of the combined group were between 0.08–0.81, with statistical significance for almost all values. Additionally, *FRS1* (I have close friends who love learning and exploring using their abilities) to *FRS2* (I have close friends who choose a learning approach that is suitable for their abilities) was the strongest (0.81).

Moreover, various studies have stated that data normality is usually assessed using skewness and kurtosis *p*-values (Pimdee, 2020). Curran et al. (1996) state that the results become suspect when univariate values approach 2.0 for skewness and 7.0 for kurtoses. Bono et al. (2019) also add that data distribution assessment usually includes a skewness and kurtosis test, with kurtosis typically done first (Table 6). After examination, recommend acceptable values for kurtosis ≤ |7| and skewness ≤ |2| (Daengneam et al., 2023, p. 82). Thus, the skewness values of −0.30 to −1.24 are acceptable, as are the Kurtosis values of 0.03 to 2.50, indicating the CFA’s appropriateness.

### 4.5. Goodness-of-fit (GoF) analysis results

The validity and influence of the causal relationship model of self-learning behavior of student-teachers were analyzed using LISREL 9.1 and the statistical criterion to measure the GoF statistics as detailed in Table 5. According to researchers, a GoF is useful when doing a structural equation model (SEM) as it helps measure how well a given data set fits a predetermined model.

Standard fit indices and criteria used in statistical programs such as LISREL 9.1 use RMSEA (≤0.05), CFI (≥0.90), SRMR (≤0.05), Chi-square *χ*^2^ (*p* ≥ 0.05), the degrees of freedom (df), as well as significance values (Koyuncu and Kilic, 2019). Alavi et al. (2020) have also suggested that smaller Chi-square values relative to the df (*χ*^2^/df ≤ 2.00) show a good fit. Other studies have reported acceptable values for GFI ≥ 0.90, AGFI ≥0.90, NFI ≥ 0.90, and RMR ≤ 0.05 (Binheem et al., 2021). Thus, from the following values, t*he CFA was found to be consistent with all the indices criteria with χ*^2^ *=* 0.63*, χ*^2^*/df =* 0.96*, RMR = 0.03, SRMR = 0.03, NFI = 0.99, and CFI = 1.00, RMSEA = 0.00, GFI = 0.97, AGFI = 0.95.* Therefore, based on the LISREL 9.1 GoF criteria and these results, it can be concluded that the model’s fit was excellent.

### 4.6. Decomposition effect results

Table 7 shows that all the model’s causal variables positively affected SDL, which, when combined, have an *R*^2^ of 51% on SDL. Also noteworthy is the TE strength for *students* on *SDL* (63%) and the TE strength for the *teachers’* effect on their *university* (61%). Also, somewhat importantly, the student-teachers perceived almost no importance in their university’s effect on their SDL (01%).

**Table 7:** Criteria, theory, and results for the GOF appraisal.

Criteria index	Criteria	Theory support	Values	Results
Chi-square: *χ*^2^	*p* ≥ 0.05	Pimdee (2020, 2021)	0.63	Passed
Relative Chi-square: *χ*^2^/df	≤2.00	Byrne et al. (2013), Pimdee (2020, 2021)	0.96	Passed
RMSEA	≤0.05	Hu and Bentler (1999)	0.00	Passed
GFI	≥0.90	Jöreskog et al. (2016)	0.97	Passed
AGFI	≥0.90	Hooper et al. (2008)	0.95	Passed
RMR	≤0.05	Hu and Bentler (1999)	0.03	Passed
SRMR	≤0.05	Hu and Bentler (1999)	0.03	Passed
NFI	≥0.90	Whittaker and Schumacker (2022)	0.99	Passed
CFI	≥0.90	Whittaker and Schumacker (2022)	1.00	Passed

**Figure 1:**
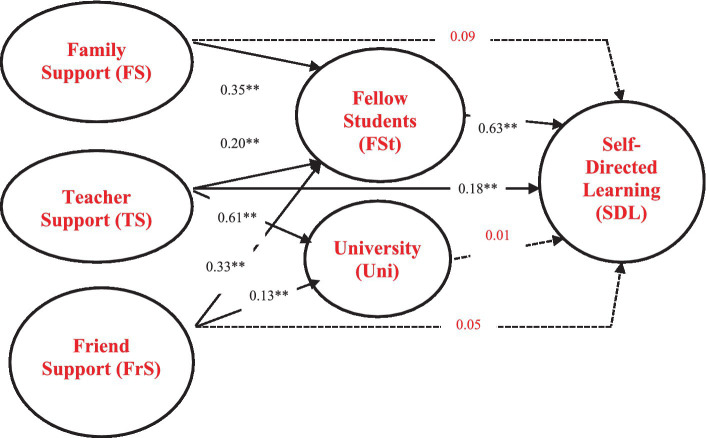
The final causal relationship model.

Table 8’s hypotheses testing results summary and Figure 1 explain that “From the hypothesis test by testing the direct influence between variables in the model, it was found that this research accepted most of the hypotheses (6 out of 10 hypotheses) at a statistically significant level of 0.01 where the cause variable had a positive influence on the effect variable.

**Table 8:** Decomposition of direct (DE), indirect (IE), and total (TE) effects of the SEM.

Path	DE	IE	TE	*R*^2^
Self-directed learning (SDL)				0.51
Family support → SDL	0.09	0.22**	0.31**	
Teacher support → SDL	0.18**	0.14**	0.32**	
Friend support → SDL	0.05	0.21**	0.26**	
Fellow students → SDL	0.63**	–	0.63**	
University → SDL	0.01	–	0.01	
Students				0.53
Family support → Fellow students	0.35**	–	0.35**	
Teacher support → Fellow students	0.20**	–	0.20**	
Friend support → Fellow students	0.33**	–	0.33**	
University				0.46
Teacher support → University	0.61**	–	0.61**	
Friend support → University	0.13**	–	0.13**	

*p ≤ 0.05, **p ≤ 0.01.

### 4.7. Hypotheses testing results

Table 8 and Figure 1 detail the hypotheses testing results. Seven of the ten hypotheses were determined to be supported.

The SDL model was determined to be consistent with the empirical data, with all the component weights being positive. Also, when the SDL model’s five aspects were ranked by total effect (TE) influence, fellow students (FSt), teacher support (TS), family support (FS), friend support (FrS), and their university (Uni) were judged most important with TE values of 0.63, 0.32, 0.31, 0.26, and 0.01, respectively. The study also determined that all the causal variables in the model had a positive influence, which is explained by the variance of influencing factors on SDL (coefficient of determination or *R*^2^) was 51% (Reisinger, 1997). Finally, seven of the ten hypotheses were determined to be supported.

These findings were consistent with other recent student-teacher SDL studies. In one, Pimdee et al. (2023) found three significant aspects of student-teacher SDL competency. When ranked in importance, these were self-control, learning desire, and self-management. Notably, in the SDL competency study, 60.90% of the student-teachers indicated their own SDL skills were acquired from social media. In another study from Australia in which Kent (2016) noted the importance of Facebook’s use in supplementing the school’s learning management system (LMS) and online learning, thus enhancing SDL skills. The authors also reported that Facebook significantly enhanced learner activity levels in classroom discussion forums. Mentz et al. (2019) also investigated social media use as an open educational resource (OERs) and reported that it had become an essential tool in SDL. Long (2000) has also added that SDL captures three psychological characteristics: motivation, metacognition, and self-control. These are related to an additional four psychological dimensions, including the 4Cs of confidence, choice, control, and competence.

### 4.8. Family support (FS)

Results from H1’s analysis of the *family’s support* influence on *SDL* were deemed unsupported (*r* = 0.09, the *t*-test value = 1.64). Moreover, the total effect (TE) for this relationship was also determined to be moderate (*r* = 0.31, *p* ≤ 0.01).

However, H2’s analysis of the relationship between the *family’s support* to *SDL* showed a moderate and favorable outcome (*r* = 0.35, *t*-test = 6.12, *p* ≤ 0.01). Additionally, the analysis of the observed variables (Table 9) showed that parents play an essential role in encouraging their children to solve problems using their knowledge and abilities (FS2) (mean = 4.20, SD = 0.70). Quite expectantly, the opposite was marked lowest when the students were asked if their parents guided them in choosing their learning methods (FS3) (mean = 3.99, SD = 0.83).

**Table 9:** Hypotheses testing results summary.

Hypotheses	Coef.	*t*-test	Accept
*H1*: Family support influences self-directed learning in a direct and positive manner.	0.09	1.64	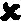
*H2*: Family support influences fellow students in a direct and positive manner.	0.35	6.12**	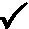
*H3*: Teacher support influences fellow students in a direct and positive manner.	0.20	4.11**	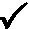
*H4*: Teacher support influences the university in a direct and positive manner.	0.61	10.57**	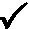
*H5*: Teacher support influences self-directed learning in a direct and positive manner.	0.18	3.15**	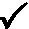
*H6*: Friend support influences students in a direct and positive manner.	0.33	6.39**	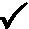
*H7*: Friend support influences the university in a direct and positive manner.	0.13	2.67**	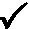
*H8*: Friend support influences self-directed learning in a direct and positive manner.	0.05	1.07	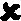
*H9*: The university influences self-directed learning in a direct and positive manner.	0.01	0.12	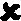
*H10*: Fellow students influence self-directed learning in a direct and positive manner.	0.63	9.34**	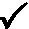

**Sig. ≤0.01, coef., correlation coefficient.

These findings are consistent with other studies which have determined that multiple external factors have an influence on learning engagement and SDL. These include the school’s support, the family’s support, and the external environment (Garciareid et al., 2015; Mazurenko and Hearld, 2015; Kelly and Zhang, 2016). Also, Elliot et al. (2017) has added that a student’s family support which can include such things as their socioeconomic status, parental support, parental expectations, family social and material resources, affects the development of learning competencies and learning motivation.

Similarly in China, Gao et al. (2021) determined that family support made major contributions to college student e-learning engagement. The authors also stated that when greater family interest is shown, learning engagement increases.

### 4.9. Teacher support (TS)

Results from H3’s analysis of the influence of *teacher support* on *fellow students* were determined to be weak but positive (*r* = 0.20, *t*-test = 4.11, *p* ≤ 0.01). Moreover, the total effect (TE) for this relationship was also determined to be weak (*r* = 0.20, *p* ≤ 0.01), as was the relationship from teachers to SDL (H5) (*r* = 0.18, *t*-test = 3.15, *p* ≤ 0.01). However, there was a very strong and positive relationship between *teachers*’ *support* effect on their *university* (H4) (*r* = 0.61, *t*-test = 10.57, *p* ≤ 0.01).

These findings are consistent with other studies in which teachers have been stated crucial in supporting and guiding students in their SDL journey. At the same time, technology can provide students with the tools and resources to take ownership of their learning and reach their full potential. According to Johns (2018) teachers need to reflect on how well their goals reached their students. The author also believes that students should be given the opportunity to make authentic choices and take ownership over their learning.

Also, from the analysis of the observed variables in Table 10, there were surprisingly low opinions given by the student-teachers on the ability of their teachers to influence all four of the aspects asked about. This is probably because some of the student-teachers who answered the questionnaire were only in their first or second years of study and were studying online during the COVID epidemic. As such, they were most probably not exposed to the normal benefits of face-to-face and classroom contact where teachers provided assistance in how to assess their learning progress. Also, the normal teacher-student encouragement process might have been weak with the creation of learning resources and teaching activities which promote SDL unavailable to the social distancing measures in place. Finally, Davis (2003) strongly commented that educators are the essential social agents in a student’s life and that they have the irreplaceable role in facilitating and directing students’ SDL.

**Table 10:** Family descriptive statistics, skewness, and kurtosis testing results.

Observed variables	Mean	SD	Level	Skew	Kurt
Family support (FS)	4.12	0.65	SWA	−0.75	1.48
My parents allow me to explore my interests and life goals (FS1).	4.16	0.73	SWA	−0.73	0.99
My parents support me in learning how to solve problems using my knowledge and abilities (FS2).	4.20	0.70	SWA	−0.90	2.05
My parents guide me in choosing a learning method suitable for my personality (FS3).	3.99	0.83	SWA	−0.89	1.29
My parents promote/support me in accessing new learning sources which match my interests (FS4).	4.12	0.83	SWA	−1.15	2.17

SWA, somewhat agree (3.50–4.49).

### 4.10. Friend support (FrS)

Results from H6’s analysis of the influence of *friend support* on *fellow students* were determined to be moderate and positive (*r* = 0.30, *t*-test = 6.39, *p* ≤ 0.01). Moreover, the total effect (TE) for this relationship was also determined to be moderate (*r* = 0.33, *p* ≤ 0.01). Additionally, the relationship in H7 from *friend support* to *university* was weak but positive (*r* = 0.13, *t*-test = 2.67, *p* ≤ 0.01). Unfortunately, H8’s hypothesis between *friend support* and *SDL* was also unsupported (*r* = 0.05, *t*-test = 1.07).

Also, from the analysis of the observed variables in Table 11, there was almost total consistency with the Likert opinion scores, with all four observed variables having a response mean of 4.02–4.06. *Friends* and their skills/talents are essential in Thai university learning but not crucial in helping student-teacher develop their SDL skills (H8 needed support).

**Table 11:** Teachers’ descriptive statistics, skewness, and kurtosis testing results.

Observed variables	Mean	SD	Level	Skew	Kurt
Teacher support	3.80	0.70	SWA	−0.56	0.60
Our teachers support our students in discovering their talents and interests (TS1).	3.83	0.81	SWA	−0.68	0.73
Teacher-initiated teaching activities promote self-learning among students (TS2).	3.84	0.79	SWA	−0.60	0.58
Teacher assignments encourage students to seek learning resources (TS3).	3.78	0.82	SWA	−0.65	0.59
Teachers advise learning how to evaluate our learning progress (TS4).	3.74	0.86	SWA	−0.61	0.49

SWA, somewhat agree (3.50–4.49).

The study’s findings concerning FrS is consistent with other studies such as Murniati et al. (2023) who determined that social media such as WhatsApp groups can empower student learning as it helps members be reminded of deadlines, assignments, and material resources. The authors also noted the importance of support from their peer groups and parents as well as the importance of having good relationships with classmates and friends.

### 4.11. Fellow students

Results from H10’s analysis of the influence of *fellow students* on *SDL* were determined to be quite strong and positive (*r* = 0.63, the t-test value = 9.34, and *p* ≤ 0.01). Moreover, the descriptive statistics analysis for *fellow students*’ observed variables (Table 12) showed there was a strong agreement for the statement that “Students know which subjects they excel in and which subjects need improvement” (FSt5) (mean = 4.25, SD = 0.67). On the other hand, each student-teacher’s learning time management is a problem according to the results from FST4 (mean = 3.91, SD = 0.74).

**Table 12:** Friends descriptive statistics, skewness, and kurtosis testing results.

Observed variables	Mean	SD	Level	Skew	Kurt
Friend support	4.03	0.74	SWA	−1.11	2.61
I have close friends who love learning and exploring using their own abilities (FrS1).	4.02	0.81	SWA	−1.00	1.84
I have close friends who choose a learning approach suitable to their abilities (FrS2).	4.03	0.80	SWA	−1.04	2.23
I have close friends who can learn on their own very well (FrS3).	4.02	0.82	SWA	−1.06	2.17
Among my friends, there is often a recommendation/exchange of new self-learning methods (FrS4).	4.06	0.87	SWA	−1.24	2.23

SWA, somewhat agree (3.50–4.49).

### 4.12. University

H9’s hypothesis testing results were unsupported (*r* = 0.01, *t*-test value = 0.12). Moreover, the total effect (TE) value between the university and SDL was extremely low (TE = 0.01). Moreover, it should be noted that from the descriptive statistics analysis of the observed variables for the university, the student-teachers felt that there was only a ‘moderate’ amount of ability to choose their course (Uni3) (mean = 3.46, SD = 1.02) (Table 13). This is probably because some of the student-teachers who answered the questionnaire were only in their first or second years of study and not aware of information about the faculty/institution’s permission to choose courses according to their interests and aptitudes, creating an environment that facilitates the discovery of new learning resources and learning methods and activities to promote self-learning of students on a regular basis.

**Table 13:** Students’ descriptive statistics, skewness, and kurtosis testing results.

Observed variables	Mean	SD	Level	Skew	Kurt
Fellow students	4.07	0.54	SWA	−0.39	1.66
Students set appropriate learning goals for themselves (FSt1).	4.08	0.69	SWA	−0.57	1.13
Students plan their studies to achieve their goals (FSt2).	4.06	0.67	SWA	−0.37	0.53
Students can evaluate the accuracy and reliability of information, news, and knowledge (FSt3).	4.06	0.65	SWA	−0.30	0.46
Students can manage their learning time well (FSt4).	3.91	0.74	SWA	−0.50	0.82
Students know which subjects they excel in and which need improvement (FSt5).	4.25	0.67	SWA	−0.77	1.59

SWA, somewhat agree (3.50–4.49).

In other studies, concerning SDL use, 87% of the Canadian faculty teachers surveyed reported that they did not favor SDL in a higher education classroom, as SDL was perceived by them as an adult education concept (Wilcox, 1996).

Table 14 shows the results of SDL’s observed variable testing. Results indicated that the most important to the student-teachers was the ability to maintain self-control (mean = 4.10, SD 0.52). This was followed by their desire for learning (mean = 4.07, SD = 0.55). Finally, they viewed their self-management skills as least important (mean 3.87, SD = 0.53).

**Table 14:** University descriptive statistics, skewness, and kurtosis testing results.

Observed variables	Mean	SD	Level	Skew	Kurt
University	3.52	0.80	SWA	−0.64	0.45
My faculty/institution organizes activities regularly to promote self-directed student learning (Uni1).	3.58	0.84	SWA	−0.49	0.41
My faculty/institution creates an environment that facilitates students’ search for learning resources and new learning methods (Uni2).	3.51	0.97	SWA	−0.58	0.03
My faculty/institution allows students to choose courses based on their interests and aptitudes (Uni3).	3.46	1.02	MOA	−0.69	0.10

SWA, somewhat agree (3.50–4.49); MOA, moderate agreement (2.50–3.49).

SWA, somewhat agree (3.50–4.49); self-directed learning (SDL).

These findings are consistent with an SDL study of 501 middle-school students in Eastern China, where An et al. (2022, p. 1) determined that six significant factors were affecting SDL. These were learning content autonomy, time management, learning strategies, learning processes, learning outcomes evaluation and reinforcement, and control over the learning environment. The authors further suggested that SDL finds inspiration from a student’s positive emotions and technological self-efficacy (Table 15).

**Table 15:** SDL descriptive statistics, skewness, and kurtosis testing results.

Observed variables	Mean	SD	Level	Skewness	Kurtosis
Self-directed learning	4.01	0.46	SWA	−0.48	3.43
Self-management	3.87	0.53	SWA	−0.37	2.26
Self-control	4.10	0.52	SWA	−0.55	2.50
Desire for learning	4.07	0.55	SWA	−0.40	1.75

SWA, somewhat agree (3.50–4.49).

## 5. Conclusion

In conclusion, this study successfully developed a model demonstrating the causal relationship between factors influencing Thai student-teacher self-directed learning (SDL), using the Theory of Planned Behavior (TPB) to investigate the role of family, teachers, friends, fellow students, and the university.

The research used a sample of 468 student-teachers from five academic majors, and the data were analyzed using path analysis conducted in LISREL 9.10. The results revealed that all the causal variables in the model positively influenced SDL, explaining the variance of influencing factors on SDL (*R*^2^) at 51%. SDL comprised five variables, when ranked in order of importance these were fellow *students, teacher support, family support, friend support*, and their *university*, with total effect (TE) influence values of 0.63, 0.32, 0.31, 0.26, and 0.01, respectively.

Also, the results revealed that the external factors of teacher support (TS) had a direct influence on SDL as well as fellow students (FSt) directly influencing SDL. It was also found that fellow students (FSt) mediated the relationships between the external factors of family support (FS), friend support (FrS), and teacher support (TS). This was especially true for students who received more support from their family, friends and teachers had better self-directed learning. This study highlights the importance of students as a mediating mechanism for the relationships between family, friends and teacher and self-directed learning.

This study also highlights issues related to each student-teachers time management ability and their perception of the university’s unwillingness to allow them to choose their courses. This study contributes significantly to the literature by explicitly investigating how TPB intrinsic and extrinsic factors impact a university student’s self-directed learning.

## 6. Limitations and suggestions

The study was limited in that the study was conducted at only one Thai university near the capital city of Bangkok. A similar study in another university from another region might generate different results. Furthermore, the study was limited to only five factors that the authors assessed were important to student-teacher self-directed learning. For future studies, other factors should be investigated.

## 7. Implications for future research

Students who receive more support from family, friends and teachers have better self-directed learning. Moreover, the study highlighted the importance of students as a mediating mechanism for the relationship between family, friends and teacher and self-directed learning. In the future, other studies might choose variables resulting from the integration of other SDL concepts or theories. Other variables may affect SDL. These include multiple groups that differ according to gender, religion, and socioeconomic status.

## Data availability statement

The original contributions presented in the study are included in the article/supplementary material, further inquiries can be directed to the corresponding author.

## Author contributions

AS and PP: conceptualization and methodology. PP and PL: software and writing—original draft preparation. AS and PL: validation. PL and WK: formal analysis and visualization. WK and TK: investigation and data curation. PP and TK: resources. AS and TK: writing—review and editing. TK and AS: supervision. AS: project administration. PP: funding acquisition. All authors have read and agreed to the published version of the manuscript.

## Funding

This research was supported by a research grant from the School of Industrial Education and Technology, King Mongkut’s Institute of Technology Ladkrabang (KMITL), Bangkok, Thailand.

## Conflict of interest

The authors declare that the research was conducted in the absence of any commercial or financial relationships that could be construed as a potential conflict of interest.

## Publisher’s note

All claims expressed in this article are solely those of the authors and do not necessarily represent those of their affiliated organizations, or those of the publisher, the editors and the reviewers. Any product that may be evaluated in this article, or claim that may be made by its manufacturer, is not guaranteed or endorsed by the publisher.
